# Assessment of the fetal lungs in utero

**DOI:** 10.1016/j.ajogmf.2022.100693

**Published:** 2022-09

**Authors:** Carla L. Avena-Zampieri, Jana Hutter, Mary Rutherford, Anna Milan, Megan Hall, Alexia Egloff, David F.A. Lloyd, Surabhi Nanda, Anne Greenough, Lisa Story

**Affiliations:** 1 Department of Women and Children's Health, King's College London, London, United Kingdom; 2 Centre for the Developing Brain, King's College London, London, United Kingdom; 3 Neonatal Unit, Guy's and St Thomas’ National Health Service Foundation Trust, London, United Kingdom; 4 Fetal Medicine Unit, Guy's and St Thomas’ National Health Service Foundation Trust, London, United Kingdom; 5 Neonatal Unit, King's College Hospital, London, United Kingdom; 6 Asthma UK Centre in Allergic Mechanisms of Asthma, King's College London, London, United Kingdom; 7 National Institute for Health and Care Research Biomedical Research Centre, Guy's & St Thomas National Health Service Foundation Trust and King's College London, London, United Kingdom

**Keywords:** fetal, magnetic resonance imaging, pulmonary development, ultrasound

## Abstract

Antenatal diagnosis of abnormal pulmonary development has improved significantly over recent years because of progress in imaging techniques. Two-dimensional ultrasound is the mainstay of investigation of pulmonary pathology during pregnancy, providing good prognostication in conditions such as congenital diaphragmatic hernia; however, it is less validated in other high-risk groups such as those with congenital pulmonary airway malformation or preterm premature rupture of membranes. Three-dimensional assessment of lung volume and size is now possible using ultrasound or magnetic resonance imaging; however, the use of these techniques is still limited because of unpredictable fetal motion, and such tools have also been inadequately validated in high-risk populations other than those with congenital diaphragmatic hernia. The advent of advanced, functional magnetic resonance imaging techniques such as diffusion and T2* imaging, and the development of postprocessing pipelines that facilitate motion correction, have enabled not only more accurate evaluation of pulmonary size, but also assessment of tissue microstructure and perfusion. In the future, fetal magnetic resonance imaging may have an increasing role in the prognostication of pulmonary abnormalities and in monitoring current and future antenatal therapies to enhance lung development. This review aims to examine the current imaging methods available for assessment of antenatal lung development and to outline possible future directions.

## Introduction

Fetal lung abnormalities are a heterogeneous group of disorders accounting for approximately 18% of all congenital abnormalities.^1^ They encompass both primary lung abnormalities and extrapulmonary conditions that detrimentally affect pulmonary growth and subsequent function. The latter group includes disorders of the renal, cardiovascular, or neurologic systems and acquired pregnancy complications such as preterm premature rupture of membranes (PPROM). Accurate antenatal diagnosis is imperative to provide appropriate counseling and perinatal care planning. The mainstay of fetal lung assessment in clinical practice is the evaluation of pulmonary size using 2D ultrasound. Although ultrasound measurements have been extensively assessed as prognostic markers in fetuses with congenital diaphragmatic hernia (CDH),^2^^,^^3^ their utility in other high-risk groups (ie, congenital pulmonary airway malformation [CPAM]) is less certain. Fetal magnetic resonance imaging (MRI) is used in clinical practice in the evaluation of specific conditions such as CDH, and although lung volumes can be assessed alongside other prognostic features such as the position of the fetal liver, image acquisition is often disrupted by fetal movement. Motion-correction approaches such as deformable slice-to-volume reconstruction (DSVR) have the potential to provide a more accurate evaluation of overall lung size. Antenatal MRI can also provide an indirect assessment of tissue perfusion and microstructure via T2* and diffusion sequences, which could yield information about alveolar development.

The current review examines the most common prenatal methodologies used in antenatal assessment of some lung abnormalities both in the clinical and research setting.

## Background: normal lung development

Lung development comprises a number of stages commencing in utero and continuing into the neonatal period and beyond. Around embryologic week 3, the respiratory diverticulum emerges from the ventral wall of the foregut into the adjacent mesenchyme. Five characteristic stages of growth and maturation are described (Table 1^4^). Disruption of these stages in pulmonary development can result in long-term sequelae.

**Table 1:** Key points of the 5 stages of lung development

### Evaluation of the fetal lung

#### Ultrasound

2D ultrasound is currently the predominant imaging modality used for assessment of the fetus in clinical practice because of its widespread availability and relative low cost. The normally developing lung is of moderate echogenicity in comparison with the adjacent liver. Abnormalities can present as hypo- or hyperechogenic areas. Primary thoracic masses should be differentiated from extrapulmonary masses, such as the bowel, stomach, or liver, which can also occupy the thoracic cavity. Ultrasound findings of primary lung hypoplasia can include reduced fetal chest circumference and fetal lung-to-head ratio,^5^ and major mediastinal shift can also be observed when hypoplastic right lung is diagnosed. Assessment of the position and size of the heart is therefore imperative.

Lung size is often used as a prognostic marker for pulmonary hypoplasia and adverse neonatal outcome. Multiple 2D assessments have been evaluated (some of the most relevant are summarized in Table 2) as an indirect estimation of lung size and as a predictor of postnatal respiratory outcome. Although extensively validated in CDH, their role and feasibility in evaluating adverse outcomes in other pulmonary conditions, such as CPAM, are less clear. In addition, machine learning techniques have the potential to be clinically valuable: using algorithms such as quantusFLM, quantitative texture analysis was achieved by processing 2D ultrasound images obtained within 48 hours of delivery. Indeed, this technique helped with the antenatal prediction of neonatal respiratory morbidity because quantusFLM was able to automatically identify and segment a region of interest in the fetal lung from 790 ultrasound images obtained from patients enrolled in specific ultrasound protocols, one of which involved the evaluation of fetal lung maturity. From this, a neonatal respiratory morbidity risk score was derived with an accuracy of 91.5% compared with using a manual delineation of the lung or only using gestational age as a predictor of adverse outcomes.^6^

**Table 2:** Ultrasound parameters used as indirect evaluation of lung hypoplasia and outcomes in congenital diaphragmatic hernia and their details of measurements

Ultrasound methods	Details of measurements	Trends and associated outcomes
Lung area (2D)	• 3 different techniques: ◦ Tracing method of the limits of the lungs^90^, ^91^, ^92^, ^93^, ^94^ ◦ Longest diameter × longest perpendicular diameter^92^, ^93^, ^94^ ◦ Anteroposterior diameter of the lung at the midclavicular line × perpendicular diameter at the midpoint of the anteroposterior diameter^92^, ^93^, ^94^	Lung area overall shown to increase over gestation regardless of the technique used in normal fetuses.^92^ In 650 normal fetuses, a linear 16-fold increase over gestation was observed from 12–32 wk, with the manual tracing method being the most reproducible method.^92^ A slight decrease in lung area can be observed after 40 wk when recorded from 60 normal singleton pregnancies at 20–40 wk of gestation using the tracing method.^93^^.^ In fetuses with CDH, the most reproducible measurement of fetal lung area was found to be manual tracing^96^over the other 2 methods.
CC	• Several measurements have been evaluated: ◦ Transverse section of the fetal chest at right angles to the fetal spine compared with the width of the heart at the 4-chamber view^95^ ◦ Tracing method along the diaphragm and inner chest walls to the apex of the lung in parasagittal section of the left chest in the midclavicular line^95^^,^^97^ ◦ Width of 1 rib calculated from average distance of 4 ribs and interspaces in section perpendicular to the long axis of the lowest 5 ribs^95^	A linear relationship was observed from scattergrams developed for each measurement between chest growth and GA between 24 and 39 wk in 83 normal fetuses.^95^ Using the transverse-section method, a flattening growth in the third trimester was observed when recorded from 610 heathy fetuses between 12 and 41 wk gestation.^97^ If CC is recorded as within the 95th percentile confidence interval, it is associated with higher chances of survival. The other methods were not described in detail.
CVR	• The congenital pulmonary airway malformation volume is estimated using the formula for a prolate ellipse ◦ CVR=(length × height × width × 0.52)/head circumference^98^	A significant growth in mean CVR in fetuses (20–35 wk) with no hydrops was recorded between 20 and 25 wk gestation, after which a progressive decrease was observed with advancing GA. Fetuses with a higher CVR are at higher risk of developing hydrops and adverse outcomes.^98^ CVR ≥1.6: early predictor of fetal hydrops
Lung span to hemithorax diameter ratio	• Lung span to fetal hemithorax ratio determined in transverse or longitudinal views: ◦ From the hilum of the lung to its outermost edge or from the hilum of the lung to the thoracic wall^99^^,^^71^	The ratio obtained from 9 fetuses with significant pleural effusions at 18–30 wk gestation seemed to decrease with GA. The ratios established were predictive of adverse outcomes, including hypoplastic lungs and severe hydrops.^71^ Lung-thoracic ratio: 44% to 77%: associated with severe hydrops and hypoplastic lungs, pleural effusions Not assessed in healthy fetuses.
CA minus HA	◦ Transverse section of the chest at level of 4-chamber view, with the heart in ventricular diastole^59^: 9Ratios were also established from these measurements^59^: ◦ CA/HA (CA-HA) × 100/CA	181 uncomplicated pregnancies between 16 and 40 wk of gestation were evaluated for both ratios^59^: CA/HA displayed a linear relationship with GA (CA-HA) × 100/CA displayed a slight decrease over GA Patients with preterm premature rupture of membranes exhibited lower mean ratios than controls, which were predictive of lethal pulmonary hypoplasia when below the fifth percentile.^59^
CC-to-AC ratio	• CC measured in the transverse plane of the fetus at the level of the 4-chamber view of the heart^100^ • AC measured in the transverse plane at the level of the stomach^86^^,^^101^	In healthy fetuses the ratio has been reported to remain constant throughout pregnancy^102^ and accurate in predicting fetal lung growth. A progressive decline of the ratio over GA was recorded, preceding the development of pulmonary hypoplasia.^100^ The fetuses that later developed pulmonary hypoplasia were also confirmed to have lower CC/AC ratios than healthy fetuses.^86^ A ratio <0.6 suggests a perinatal lethal disorder.
LHR	• LHR ◦ Longest diameter method: product of the longest 2 perpendicular linear measurements of the lung, measured at the level of the 4-chamber view of the heart^92^^,^^103^ ◦ Tracing method: tracing the limits of the contralateral lung^93^ divided by the head circumference to obtain the LHR	1. a) The longest diameter method displayed an LHR increase over GA in healthy fetuses imaged from 12–32 weeks, but was deemed least reproducible and as overestimating the lung areas.^92^ b) When evaluated in fetuses with CDH, the ratio was significantly lower in nonsurvivors than in survivors.^103^ 2. a) Using the tracing method, left and right LHRs were established for 60 normal singleton pregnancies at 20–40 wk of gestation, both displaying an increase until 20 wk and a slight decrease at 40 wk^93^ b) When evaluated in fetuses with CDH at 22–28 wk gestation, the tracing method was the most reproducible measurement, and the LHR was significantly lower than in normal lung development and was one of the most significant predictors of survival.^104^^,^^105^ • <25%: disease classified as severe • 26%–45%: disease classified as moderate • >45%: disease classified as mild
Observed to expected LHR (o/e LHR)	• o/e LHR × 100 ◦ Measured LHR expressed as a percentage of the expected normal mean for GA^40^^,^^105^	o/e LHR is expressed as a percentage of the expected mean for GA (ultimately determined to be dependent on GA^107^) and was found to be a useful predictor of subsequent survival, with higher values correlating with an increased rate of survival.^108^ Conversely, very low values were associated with extreme pulmonary hypoplasia and low survival rates.^40^ • <15%: virtually no chance of survival: extreme pulmonary hypoplasia • 15%–25%: predicted survival ≈ 15%: severe pulmonary hypoplasia • 26%–45%: predicted survival 30%–75%: moderate pulmonary hypoplasia • >45%: very likely to survive: mild pulmonary hypoplasia
QLI	• QLI^41^=lung area/(head circumference/10)^2^	This index seems to be more stable throughout gestation (can be assessed independently from GA) and might predict neonatal survival more accurately than LHR.^109^ From retrospective data, QLI seems to have a relatively constant value between 16 and 32 wk.^41^ This index also has the potential to quantitatively determine which CDH fetuses would benefit the most from surgery (ie, tracheal occlusion).^109^ • <0.3: virtually no chance of survival • 0.6: poor outcome and associated with small lungs • >0.89: higher chance of survival
Lung to thorax transverse area ratio	• Area of bilateral lung profiles divided by the profile area of thorax at the level of the 4-chamber view of the heart^110^	No significant relationship was found between the ratio and GA in normal fetuses and CDH cases.^110^ The ratio was lower in cases with CDH than in normal fetuses, and was found to accurately characterize respiratory outcome and to be well related to the postoperative respiratory condition.^110^ • Normal range: mean ratio 0.52±0.04 • Cases with CDH: mean ratio 0.24±0.08

AC, abdominal circumference; CA, chest area; CC, chest circumference; CDH, congenital diaphragmatic hernia; CVR, congenital pulmonary airway malformation volume ratio; GA, gestational age; HA, heart area; LHR, lung-to-head ratio; QLI, Quantitative lung index.

Although 3D sonography has been assessed in research settings to evaluate lung volumetry, it is not used routinely in clinical practice because it is too time-consuming and has not been shown to be an accurate predictive tool. Using the conventional multiplanar mode allows simultaneous display of 3 perpendicular views of the lung. Pulmonary volume calculated using this technique has been found to be reproducible in both normal pregnancies and in fetuses at high risk of pulmonary hypoplasia (including in cases of PPROM), fetuses with suspected intrauterine growth restriction, and those with congenital anomalies.^7^ The evolution of a virtual organ computer‐aided analysis (VOCAL) has allowed the fetal lung volume to be calculated as a rotational measurement around a fixed axis, and reference values of both lungs have been established.^8^ VOCAL has a shorter acquisition time than the multiplanar technique when performed between 20 and 37 weeks of pregnancy,^9^ and provides good prediction of pulmonary hypoplasia severity when compared with postmortem volumes.^10^ However, VOCAL is not commonly used in clinical practice because it is highly dependent on fetal position, is sensitive to both motion and acoustic shadows, and requires significant training. Although some studies have demonstrated good intra- and interobserver variability (mean difference for right lung/left lung, respectively: VOCAL, −2.09/−2.09 and multiplanar, −0.93/−0.59) using the 2 techniques,^7^ others have indicated that VOCAL has higher interobserver variability and lower interobserver agreement compared with the multiplanar technique.

#### Magnetic resonance imaging

**Figure 1:**
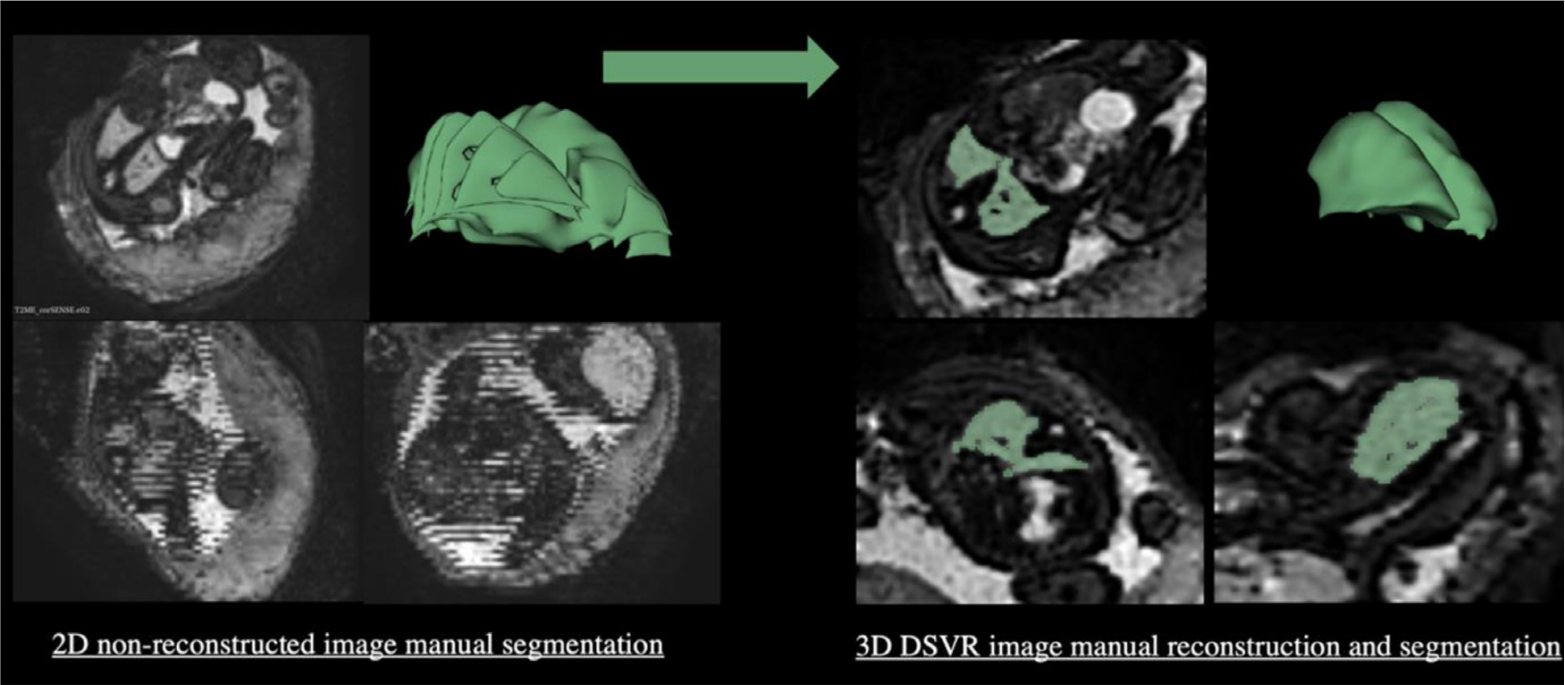
3T images of low-risk fetus at 30 weeks before and after applying DSVR^17^,^18^ DSVR: Deformabale slice-to-volume registration.

MRI has been increasingly used for assessment of fetal anatomy since the advent of ultrafast sequences in the 1990s. It is noninvasive and provides excellent soft-tissue contrast and high spatial resolution with a large field of view. It is also less restricted by factors such as maternal habitus, fetal lie, and oligohydramnios compared with ultrasound. The fetal lungs, trachea, and bronchial tree have a high water content in utero and high signal intensity on T2-weighted images, facilitating both morphologic and volumetric assessment. In a series of 74 fetuses with ultrasound-suspected thoracic abnormalities, MRI assessment led to a change in management in 8% of cases, namely either termination of pregnancy or altered care or birthing plans. MRI was indeed able to provide precise additional information in those cases, such as clearer delineation of ultrasound-detected masses for example, which helped establish the severity of the diagnosis.^11^ Other MRI parameters have also been evaluated, including the lung-to-liver signal intensity ratio as a predictor of neonatal survival, but this has been restricted to conditions resulting in hypoplastic lungs and is lacking significant results in other high-risk groups.^12^ 3D MRI-derived lung volumes have been evaluated as a prognostic marker for pulmonary hypoplasia in CDH^13^^,^^14^ and are used in clinical practice; however, validation in other high-risk groups is limited and confined to the research setting.^15^^,^^16^ Recent advances in image acquisition such as fast single-shot, accelerated MRI sequences and postprocessing techniques such as DSVR^17^ now yield reconstructed high-resolution motion-corrected 3D volumes from multiple stacks of slices affected by nonrigid motion (Figure 1). DSVR oversamples the region of interest in which a single stack is selected as a template and iteratively aligns the reconstructed volume to each individual slice in a common coordinate space thereby accounting for unpredictable motion. It can potentially be applied to several types of images with different protocol acquisition and help determine lung volumes in any high-risk group.^18^ Patch-to-volume reconstruction has also been developed for use in more mobile structures and has proven successful for reconstructions of the fetal body and placenta; however, it has not been fully assessed for lung reconstruction to date.^19^ Fetal body volumes have also been calculated from MRI data to create lung-to-body volume ratios to account for the overall size of the fetus. Indeed, at later gestational age there is a wider variability in lung size correlating with the total volume of the fetus. This technique has been used in uncomplicated pregnancies,^20^ those at high risk of preterm birth,^21^ and fetuses with CDH.^14^

Avena-Zampieri. Assessment of the fetal lungs in utero. Am J Obstet Gynecol MFM 2022.

**Figure 2:**
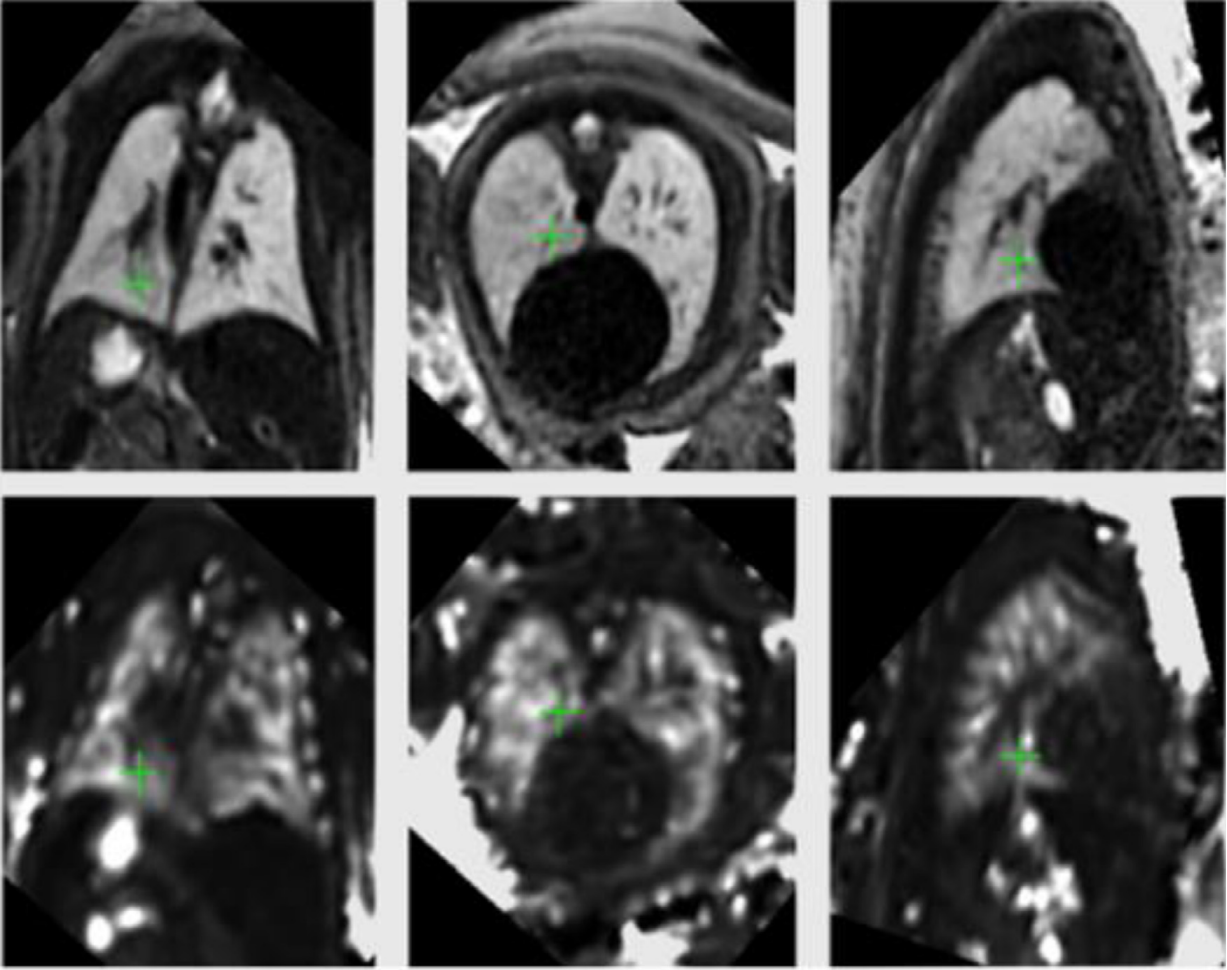
T2 (top) and T2* (bottom) aligned maps of a healthy fetus at 35 weeks’ gestation scanned on a 3T MRI scanner *3T MRI*, 3 Tesla magnetic resonance imaging.

In addition to anatomic postprocessing pipelines, the use of multiparametric MRI techniques, including combined diffusion and relaxometry,^22^ is a rapidly evolving field in the research setting and has the potential to simultaneously describe intrinsic lung characteristics such as microstructure and tissue perfusion, which may provide an indirect assessment of lung maturity. Diffusion MRI evaluates the microstructure of tissue and extracellular fluid spaces on the basis of signal loss because of molecular motion of water. This has the potential to provide information on alveolar and parenchymal development. Quantitative assessment can be achieved by obtaining several derived measurements including the apparent diffusion coefficient (ADC) (this is reduced in the presence of increased cellular complexity). ADC values have been shown to increase in the lungs of healthy fetuses^23^ as gestation continues, which may reflect development. T2* imaging has the potential to identify alterations in tissue oxygenation. T2* relaxation exploits the blood oxygen level–dependent effect, which works on the different paramagnetic properties of oxygenated and deoxygenated hemoglobin. Quantitative T2* maps can be obtained using gradient-echo imaging with several echo times (Figure 2). Although limited, T2* relaxation times have been quantified in several fetal organs. In the fetal lungs, it seems that T2* values increase with gestation until 30 weeks, but a clear trend was hard to establish given the small number of cases studied and limited gestational age range.^24^ The increase observed may be attributable to an increase in metabolic activity across maturation. In addition, other functional MRI techniques, such as perfusion MRI, where the circulation in the vascular bed per unit volume can be recorded by evaluating indirect markers of tissue perfusion, or T1 relaxometry, assessing the relaxation of the longitudinal magnetization, have been assessed in previous studies and may play a role in pulmonary characterization in the future.

Avena-Zampieri. Assessment of the fetal lungs in utero. Am J Obstet Gynecol MFM 2022.

### Other technologies

Artificial intelligence (AI) techniques have for example been used to automate biometric measurements such as head circumference or biparietal diameter measurement. Therefore, fully convolutional neural networks combined with a regression network have been employed using multiscale information to pinpoint the edge of the skull. This results in more accurate estimation of the measurements (evaluated with 3 metrics and ranked first on the basis of absolute head circumference vs Thomas et al's^25^ automated evaluation ranked 25th) compared with that of the previous network that used a single scale to segment the head region of a fetus, and compared with current 2D ultrasound fetal head measurements.^26^ From these encouraging results, an ongoing retrospective data study is developing a software system for automatic fetal lung volume segmentation in MRI. This is based on the deep-learning 3D U-NET, a semantic segmentation network based on the convolutional neural network. Using this approach, the aim is to ultimately build an AI system able to identify patients in the antenatal period at high risk of developing CDH-associated pulmonary hypoplasia.^27^ Multimodal fusion imaging, which allows superimposition of real-time ultrasound images and multiplanar reconstruction images of MRI, is another promising technique, which has been assessed to date in multiple conditions including CDH and pulmonary sequestration.^28^

### Abnormal lung development and associated lung pathologies

Normal fetal lung development can be disrupted at any stage of development (Table 1). Prognosis is dependent on the underlying condition and gestation of the insult. Congenital abnormalities of the lung can be primary such as CPAM or secondary to a reduction in thoracic space, adversely affecting pulmonary development, such as CDH or lethal skeletal dysplasia.

#### Primary pulmonary conditions

**Figure 3:**
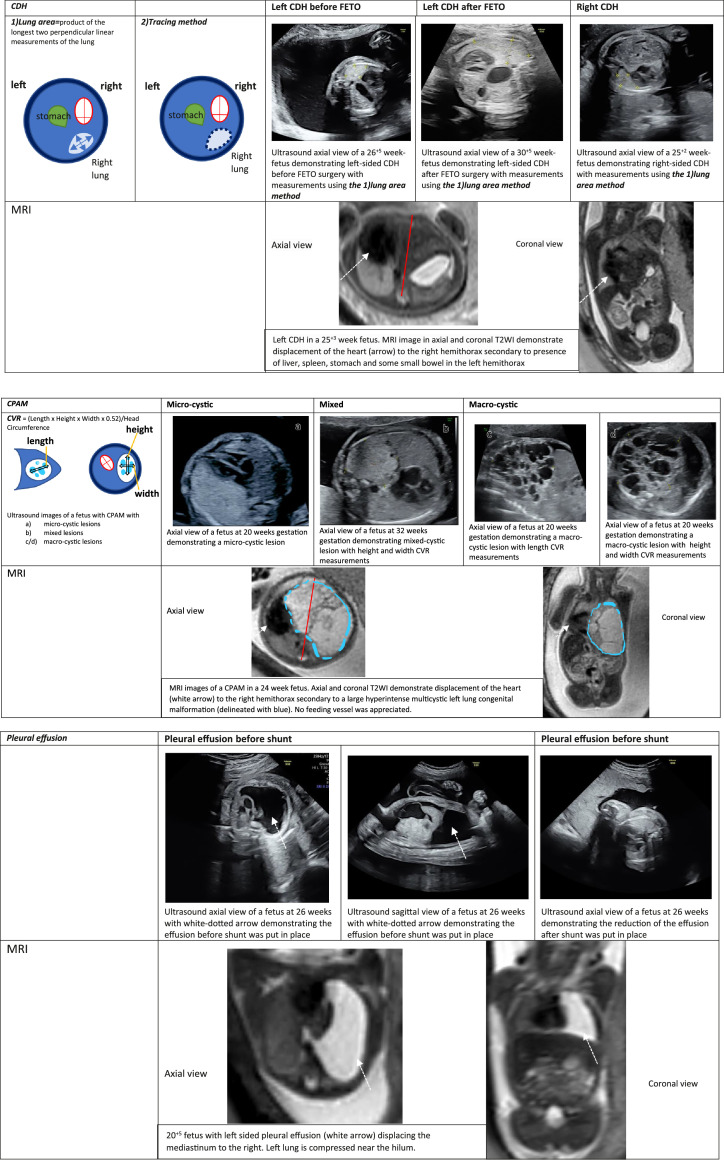
Ultrasound and MR images comparison with measurement diagrams of CDH, CPAM, and pleural effusion cases *CDH*, congenital diaphragmatic hernia; *CPAM*, congenital pulmonary airway malformation; *CVR*, congenital pulmonary airway malformation volume ratio; *FETO*, fetoscopic endoluminal tracheal occlusion; *MRI*, magnetic resonance imaging.

1. CPAM occurs because of underdevelopment of the terminal bronchiolar structures from which multiple lung cysts arise. It accounts for 30% to 40% of congenital lung malformations. A significant proportion of cases regress in the third trimester. Poor prognosis is associated with the presence of hydrops (10% of cases) and large masses.^29^ Antenatal diagnosis can be made during routine second-trimester ultrasound evaluation. Hyperechogenic lesions in the fetal chest are divided into solid or microcystic, macrocystic with ≥1 large cysts (>2 cm), and mixed with areas that are solid intermixed with areas containing multiple cysts <2 cm in diameter^30^ (Figure 3).

Avena-Zampieri. Assessment of the fetal lungs in utero. Am J Obstet Gynecol MFM 2022.
2. Bronchopulmonary sequestration (BPS) is a nonfunctional mass of lung tissue that does not communicate with the normal tracheobronchial tree. It accounts for 0.15% to 6.4% of congenital lung malformations^31^ and can be subcategorized into intralobar or extralobar sequestration. The latter is found in <25% of cases but is more commonly associated with pleural effusion. Fetal pulsed-wave Doppler during ultrasound evaluation is often used to assess for the presence of an aortic feeding vessel, the size of which can affect hemodynamic status and potentially lead to high-output cardiac failure and life-threatening perioperative hemorrhages.^32^ In several case reports, MRI has accurately diagnosed complex thoracic lesions, distinguishing CPAMs from BPS by accurately characterizing the anatomy of the feeding vessels in BPS.^33^ MRI had a higher sensitivity (71%) in determining whether a systemic feeding vessel was present, demonstrating the arterial supply and delineating the mass more clearly than ultrasound (49%).^34^ On T2-weighted MR images, high signal intensity of the lungs relative to that of normal lung tissue has been observed in sequestration lesions, and changes in vascularity in these T2-hyperintense lesions.^11^ Accurate diagnosis of CPAM and its distinction from BPS are crucial in optimizing postnatal management plans in complex cases of anomalous vessels. Laser ablation of the feeding artery can be performed antenatally using Doppler ultrasound with the aim to improve prognosis in severe forms associated with massive pleural effusions and hydrops.^35^

#### Congenital abnormalities resulting in a reduction in thoracic space

1. CDH is an anomaly with a prevalence of 1 in 4000 births.^36^ It is characterized by malformation of the diaphragm causing part of the abdominal viscera to herniate into the thorax (Figure 3). Compression of the ipsilateral lung can displace the heart and mediastinum while also affecting the contralateral lung. This compression affects lung development and can culminate in pulmonary hypoplasia. Prognosis is dependent on the severity, type, and laterality of the hernia.

On ultrasound, features may include absence or partial absence of the pleural line that represents the parietal pleura (ie, the layer of pleura associated with the walls of the pleural cavity).^37^ Other findings can include polyhydramnios, liver herniation, mediastinal shift, abnormal cardiac axis, and inability to demonstrate the normal stomach bubble.^38^ However, most of these parameters have been deemed to have insufficient sensitivity and specificity to assist clinical decision-making and are mostly applicable to fetuses with left-sided CDH.^39^ As previously discussed, it is essential to determine the location and size of abdominal organs in the thoracic cavity to confirm the diagnosis of CDH and to stratify the severity of the lesion. Various scores have been developed to support clinicians in predicting the degree of subsequent pulmonary hypoplasia using an ultrasound approach (Table 2). These include the lung-to-head ratio (LHR), which provides an estimate of the size of the lung contralateral to the lesion, normalized by the head circumference. Because the LHR changes over gestation, with the lung growing at a faster rate compared with the head, the observed/expected ratio has been introduced (o/e LHR). Here the LHR is normalized for the expected score measured in healthy fetuses at the same gestational age. The above scores based on ultrasound assessment of lung volumes have been found to offer good prediction value of neonatal outcome in CDH fetuses.^40^ Quintero et al introduced another parameter that stays constant throughout pregnancy for the assessment of lung volume, the quantitative lung index (QLI) (Table 2). In their study, a QLI <0.6 was predictive of small lungs and confirmed its accuracy in anticipating adverse neonatal outcome, especially in the CDH groups presenting an index between 0.4 and 0.6.^41^ Other parameters have been evaluated, such as the lung-to-liver signal intensity ratio studied by Yamoto et al^12^ as a marker for fetal lung maturity where correlation between increasing lung-to-liver signal intensity ratio and gestation in control fetuses was further confirmed in the 47 fetuses with CDH; however, there is limited applicability to this technique, with around one-third of diagnoses missed. Only small cohort studies have tried to quantify the degree of liver herniation to evaluate antenatal fetal lung maturity in isolated CDH cases and to predict postnatal outcome.^12^^,^^38^ To standardize the use of the aforementioned methods, a guideline has been developed by the European Reference Network for rare Inherited and Congenital Anomalies, providing a more practical guide for the assessment of isolated CDH (Table 2). Several studies have evaluated the feasibility of pulmonary vascular Doppler in predicting survival in fetuses with CDH; however, these studies vary significantly with regard to their methodology and number of participants evaluated, and all came to the conclusion of progressive reduction in intrapulmonary blood flow independently of lung size.^42^^,^^43^

MRI is increasingly being used to evaluate CDH in clinical practice because the available higher resolution, 3D continuity, and soft-tissue contrast allows for the hernia to be characterized with regard to its location, type (ie, posterolateral Bochdalek hernia, anterior Morgagni hernia, or hiatus hernia), and the position of other structures such as the liver, stomach, and bowel.^44^ In uncomplicated pregnancies there is good agreement between ultrasound and MRI-derived lung volumes (mean of difference for right lung volume/left lung volume: −2.13/−0.86)^45^; however, in cases of CDH, ultrasound underestimates the volume of the contralateral lung by 25%. This might be attributable to the smaller number of sections and planes obtained in ultrasound compared with MRI.^46^ The ipsilateral lung has been reported across several other studies to be visible in only 55% to 64% of cases using 3D ultrasound,^47^ which thus does not yield reliable measurements, whereas MRI-determined observed/expected total lung volume (o/e TLV) in 85 fetuses with an isolated left CDH with a range of 18 to 39 weeks functioned better than ultrasound in predicting survival antenatally.^48^^,^^49^ MRI also provides good prediction for the requirement of extracorporeal membrane oxygenation, and for the development of chronic lung disease in surviving infants.^50^^,^^51^ MRI also has an increasing role in assessing suitability of pregnancies complicated by CDH for surgical antenatal treatments such as fetoscopic endoluminal tracheal occlusion.^52^

In the research setting, ADC values were also found to be significantly different at both low and high b-values (reflecting the strength and timing of the gradients used to generate diffusion-weighted images) between the normal developing lungs and the ones affected by CDH, but unrelated to lung volume.^53^ This a promising technique and needs evaluation in further studies: the effect of capillary perfusion and water diffusion in the extracellular extravascular space could potentially be quantitatively obtained (reflected in those ADC values) to differentiate normal from abnormal tissue structures and be evaluated in CDH cases with different severities to predict adverse outcomes and/or need or timing for surgical intervention.

1. Skeletal dysplasia encompasses a series of >400 heterogeneous disorders affecting any or a combination of bone or cartilage development, growth, and structure.^54^^,^^55^ Many of these abnormalities result in a small or deformed thorax, increasing the risk of airway malformation and pulmonary hypoplasia, which is significantly associated with lethality.^56^ Diagnosis and subsequent pulmonary consequences have been evaluated using ultrasound parameters of thoracic-to-abdominal or thoracic-to-head-circumference ratios. The diagnostic rate of specific skeletal dysplasia subtypes was only 65% to 68%,^57^ but prediction of lethality using these parameters is very effective. Limited studies have specifically evaluated 3D ultrasound–derived lung volumes and their correlation with outcomes, one of which was able to accurately predict lethality from pulmonary hypoplasia in fetuses between 20 and 32 weeks of gestation with skeletal dysplasia by using the VOCAL 3D ultrasound method.^58^ Other 2D sonographic parameters, including chest circumference (CC) and chest circumference to abdominal circumference (AC) ratio, were assessed simultaneously with these 3D lung measurements, and were found to have lower sensitivities and specificities than total lung volume.^58^^,^^59^ Lung volumes in fetuses with skeletal dysplasia have also been assessed using MRI. In 36 fetuses with suspected skeletal dysplasia, this measurement proved superior to ultrasound by demonstrating an association with a reduction in o/e TLV, with values of 47.9% and 0.124 in FL/AC determined to be potentially useful clinical cutoffs in the prediction of lethality.^60^ MRI was also found to provide clear depiction of soft tissue, allowing the analysis of the fetal spine and differentiation between the various subphenotypes of skeletal dysplasia.^61^
2. Significant cardiomegaly puts the fetus at increased risk of impaired lung growth because of a reduction in thoracic space.^62^ In CDH fetuses, smaller branch pulmonary arteries (PAs) compared with those of control fetuses of the same gestational age have also been recorded on cross-sectional imaging through the fetal chest using ultrasound, establishing a potential correlation between PA diameter and extent of hypoplasia.^63^^,^^64^ Fetal cardiac abnormalities that can be associated with cardiomegaly include tricuspid valve regurgitation, particularly Ebstein's anomaly. There is evidence that in fetuses with right outflow obstruction, early disruption of pulmonary vascular growth alters the branching morphogenesis of the airways,^65^ as assessed using ultrasound parameters, whereas reduced pulmonary flow at a later stage of gestation alters alveolarization.^66^

Clinical diagnosis of congenital heart disease is established through routine cardiac screening using ultrasound, which rose from 40% of cases being accurately detected in England in 2013^67^ to 52% in March 2021 following the COVID-19 pandemic.^68^ Very few studies have explored the impact of fetal cardiac malformations on neonatal respiratory outcomes because of multiple confounding variables in the perinatal period and the lack of accurate diagnostic tools to differentiate between cardiac and pulmonary etiology. One study reviewed the echocardiograms of CDH fetuses and suggested that an enlarged contralateral PA diameter in the midtrimester correlates with postnatal death, which can be attributed to the possible overworking redistribution of blood flow from the left to the right part (contralateral) of the heart, and discrepancy between fetal branch PA diameters correlated directly with postnatal respiratory morbidity.^69^ Fetal cardiovascular magnetic resonance (CMR) sequences have an additional value in simultaneously assessing cardiac abnormalities and providing invaluable information about the fetal lungs. Fetal CMR has efficiently been used to study fetal circulatory physiology in fetuses with CDH, providing new insights on blood flow distribution in the fetal circulation. Providing supplementary maternal oxygen during fetal CMR can physiologically test the vessels and vasoactive responses of certain fetal organs, and could be used in the future to determine the relationship between absolute volumes of the lungs and the maturity of the arterial tree in terms of size and physiological characteristics.

1. Pleural effusion is defined as an accumulation of fluid within the pleural space (Figure 3), and is estimated to occur in 1 of 15,000 pregnancies.^70^ It can arise because of a number of different conditions such as infection or CHD, all with the potential to culminate in abnormalities in lung growth. A large pleural effusion can act as a space-occupying lesion, compressing the lungs. Effusions have additionally been found to be associated with a decreased number of cells, alveoli, and overall lung size.^71^ Diagnosis is reliably made using ultrasound, with the effusion appearing as an anechoic area surrounding the lungs.^72^ Effusions may also be associated with hydrops or mediastinal shift, which may require in utero interventions. Treatment options include drainage with pleurodesis, thoracentesis, and thoracoamniotic shunting,^73^ the last being the most effective in improving perinatal outcomes.^74^ Limited studies have used fetal MRI to assess hydrothorax and its effect on pulmonary development.^75^^,^^76^ These have however highlighted the value of MRI in better defining the underlying etiology, as demonstrated in one retrospective study conducted on 76 fetuses in which postnatal diagnosis confirmed the prenatal diagnosis in 51 of the 56 lung lesions (91%), including CPAM, BPS, and bronchopulmonary cysts.^77^

#### Conditions resulting in oligohydramnios/anhydramnios

Conditions that result in second-trimester oligohydramnios may restrict fetal lung growth and result in pulmonary hypoplasia, as shown in both animal models and human fetuses.^78^^,^^79^ This may be because of congenital abnormalities such as bilateral renal agenesis or acquired abnormalities such as PPROM.

1. Renal tract abnormalities and early-onset oligohydramnios are associated. Pulmonary hypoplasia in these cases is thought to be attributable to low urine production that results in a decrease in intraamniotic pressure and increase in the alveolar–amniotic pressure gradient. A population-based study suggested a positive correlation between pulmonary hypoplasia and oligohydramnios, in which oligohydramnios-exposed children presented an 8% higher increased admissions rate with respiratory illness than nonexposed children.^78^ The exact mechanism by which lung hypoplasia ensues is unclear, but animal models suggest that oligohydramnios at the pseudoglandular stage reduces elastin deposition, alveolarization, and the amount of collagen in the fetal lung.^80^ There are limited data specifically evaluating lung development antenatally in cases of renal agenesis, but postnatal quantitative analysis has demonstrated a reduction in size of the pulmonary airways and growth within the acinar region, with resulting hypoplastic lungs.^81^ MRI images are less affected by oligo- and anhydramnios than ultrasound images.^82^ The role of MRI in assessing pulmonary development in urinary tract abnormalities in these cases of oligo- and anhydramnios has been assessed through several studies. One study yielded images from fast gradient-echo sequences and true fast imaging analyzed by an experienced observer for valuating signal intensity of the lungs to diagnose pulmonary hypoplasia. Low signal intensity in the lungs was thus identified in a fetus with Meckel–Gruber syndrome. Overall, MRI was deemed superior in detecting the bilateralism and severity of the renal diseases, and denoted better visualization of the fetal whole body in a single cut compared with ultrasound. Reaching a diagnostic accuracy of 96% for urinary tract anomalies in this study, MRI demonstrated its potential in characterizing the associated extrarenal fetal anomalies, such as pulmonary anomalies, with high accuracy.^82^

Neonates with second-trimester PPROM have an increased risk of pulmonary hypoplasia (≥47%)^83^ and other respiratory morbidity, including pulmonary hypertension and pneumothoraces.^84^ Gestational age at the time of membrane rupture was found to be the best predictor of pulmonary hypoplasia (superior to the duration and degree of oligohydramnios^85^).

Ultrasound-derived CC, CC-to-AC ratios,^86^ and CC-to-femur-length ratios^5^ have low sensitivity in predicting adverse outcome in PPROM. Fetal breathing, fetal movements, and thoracic-circumference-to-abdominal-circumference ratios were not found to be predictive of respiratory outcome.^87^ Overall, there is limited accuracy of imaging biometric parameters in the prediction of pulmonary hypoplasia in midtrimester PPROM,^88^ but some recent evaluations of o/e LHR and QLI in live birth with PPROM occurring before 24 weeks of gestation proved valuable in predicting lethality.^89^ Moreover, lower-chest-area-to-heart-area mean ratios were established to be lower than those of controls, and predictive of lethal pulmonary hypoplasia when below the fifth percentile.^59^

MRI has been used to evaluate lung volumetry and its correlation with postnatal outcome in fetuses with second-trimester PPROM. Lung volumes <60% of that predicted from normal ranges were found to be incompatible with postnatal survival.^15^ Our group has also demonstrated that fetuses with high risk of preterm birth (both with and without membrane rupture) have smaller MRI-assessed lung volumes after adjustment for overall fetal size.^21^ A period of prolonged oligohydramnios during mid-gestation leads to a decrease in radial alveolar in the fetal lung, potentially mirrored by the significantly reduced signal intensities of the fetal lung tissue recorded across several studies on T2-weighted sequences. MRI therefore also has the potential to provide additional information pulmonary hypoplasia due to prolonged exposure of oligohydramnios induced by PPROM.

## Conclusions

Ultrasound provides a cost-effective, widely available tool that is effective in detecting a high percentage of lung lesions. MRI, with its ability to display multiple planes and greater tissue differentiation, can provide additional information in a number of pulmonary conditions. Although already used in the clinical setting, rapid advances in the field, particularly with regard to motion correction and functional tissue assessment, indicate its potential to provide significantly more information on lung development in the future. This may enhance not only prognostication and antenatal counseling but also monitoring of existing and future therapies.
